# Survey on general awareness, mental state and academic difficulties among students due to COVID-19 outbreak in the western regions of Uganda

**DOI:** 10.1016/j.heliyon.2020.e05454

**Published:** 2020-11-06

**Authors:** M. Abisha Meji, Milon Selvam Dennison

**Affiliations:** School of Engineering & Applied Sciences, Kampala International University (Western Campus), Uganda

**Keywords:** Psychology, Student, Academic, Mental state, Uganda, Pandemic, COVID-19, Lockdown

## Abstract

This academic research is carried out to access the general awareness, mental state and academic difficulties among different age groups of students studying in various schools, colleges, or Universities during this lockdown period due to the COVID-19 crisis in the western regions of Uganda. An aggregate of 405 students participated in this survey. Among them 253 students are from rural regions, 59 students are from semi-urban regions and 93 students are from urban regions. This survey is classified into three sections: the first section spotlights the perceptive level of students about the COVID-19 crisis, the second section emphasizes the mental state of students and the final section highlights the academic difficulties faced by the students during this lockdown period. A statistical run is deliberated with the aid of SPSS version 20 software to evaluate the significance level (P-Value<0.05) of each question among the localities.

## 1 Introduction

The worldwide community is slowing down and eventually halts due to the spread of novel Coronavirus SARS-CoV-2 (2019-nCoV). In the African continent, the infection due to the novel virus has spread in many nations (Gilbert et al., 2020). The World Health Organization (WHO) named the disease caused by 2019-nCoV as COVID-19 and has declared this as pandemic on March 11, 2020. The government and medical workers in the African territories are striving hard to control the spread rate of this pandemic. COVID-19 is negatively affecting the country's economy. The urban-based manufacturing and service sectors in the African continent accounts for 64% of Gross Domestic Product (GDP) is hit hard by this COVID-19 pandemic and it leads to significant losses in productive jobs (https://www.un.org/africarenewal/news/coronavirus/eca-economic-impact-covid-19-african-cities-likely-be-acute-through-sharp-decline-productivity). Firms and organizations in African urban areas especially small and medium enterprises which provide 80% of employment in the African continent are highly vulnerable due to the impact of COVID-19. These risks are aggravated by a possible climb in the cost of the basic needs of humans in Africa. The COVID-19 pandemic is severely testing Africa's economic, social and political resilience (Van Bavel et al., 2020; Nicola et al., 2020). Due to this pandemic, the mental state of humans especially students are highly tested for its resistance.

Researchers consider that the mental health of an individual is closely linked with the financial conditions (Shor et al., 2013). It is believed that poor financial conditions, psychological difficulties like depression, anxiety, stress, may adversely affect the mental health of an individual. Physical well-being positively affects the mental health of an individual (Prakash and Gupta, 2019). Psychological distress, in the form of anxiety and depression, is associated with serious health conditions.

Depression is one of the common mental health issues prevailing among people of different age groups, especially college students. It causes various emotional and physiological disturbances and can seriously impair the ability of an individual to perform different tasks. Any traumatic experience, a physical or psychological loss like loss of a job, death of a loved one, loss of possession, or any serious injury may cause depression. Finally, it may lead to a feeling of loneliness, sadness, loss of interest in activities of day-to-day life and even tempt the student to commit suicide (Pompili et al., 2014; Derks et al., 2017; Caspi and Moffitt, 2018).

The term anxiety is used to describe an uncomfortable and unpleasant feeling that an individual experiences in a stressed or fearful situation (Hjeltnes et al., 2018). Physiologically, it results in chronic over-arousal and a state of tension that may prepare a person for a fight or flight reaction. It is faced by students mostly because of the pressure of being in a new environment and a strong desire to perform well. Students with anxiety disorders like disturbances in mood, thinking, behaviour and physiological activity, show a passive attitude in their academics such as lack of interest in work, poor performance in examinations and a disturbed routine (Eslami et al., 2016; Parray and Kumar, 2017).

Stress is a state of being threatened and undergoes certain psychological changes that prepare one to handle the threat through sustained activity (Griep et al., 2016). The perception of stress is affected by the changes occurring in an individual's life, the ability of an individual to deal with problems and daily hassles and unpredictability of upcoming life threats (Juster et al., 2016; Heinen et al., 2017). It is not about measuring the types or frequency of stressful life events but the perception about the severity of these events. It is affected by the ability to handle such stressors. Different individuals may suffer from similar life events but perception about these events determines the experience of stress (Harkness and Monroe, 2016).

Uganda, an East African territory well known for its natural resources and called ‘Pearl of Africa’ is striving hard to sustain in all fields especially in education, agriculture, and medicine. In the case of education, it has an education structure and also the government of Uganda recognizes education as a basic human right (Wamaungo, 2017; Nzabona and Ntozi, 2017; Datzberger, 2018). Over the world, currently, there are about 1.4 billion students are pursuing their school and higher education studies (Bozkurt et al., 2020). Due to this COVID-19 pandemic, traditional classroom learning is affected drastically. To overcome this crisis many countries across the world are adopting E-learning platforms for students (Ali, 2020; Bozkurt et al., 2020). Uganda is still a developing country that is not as par with the developed countries in terms of network coverage, data package, and usage of smart gadgets. So E-learning in Uganda is a burdensome activity and even it creates academic anxiety among suburban and rural students of Uganda.

### 1.1 Study purpose

The purpose of this study is to measure the general awareness about COVID-19, mental state, and academic anxiety among the various age group of students studying in different Schools, Colleges, or Universities in the western districts of Uganda during this lockdown period due to the COVID-19 crisis. The ‘Republic of Uganda’ is classified into four regions and the western region is one among them which comprises 26 districts. The western region of Uganda has 10 recognized Universities and its associated schools. According to the Higher Education data, about 10,000 + students are studying in the above-said institutions. To this end, the research questions were as follows:

1) What is the perceptive level of students about the COVID-19 crisis?
2) What is the mental state of students during this lockdown period due to the COVID-19 pandemic?
3) What are the academic difficulties faced by the students during this lockdown period?

### 1.2 Ethical consideration

Approval to conduct this survey research is obtained from the ‘Research Innovation Consultancy and Extensions (RICE)’ board of the author's University system. Students who participated in this survey were voluntary and participation did not affect their grades. The questionnaire was coded to provide discretion and secrecy.

## 2 Methodology

**Figure 1:**
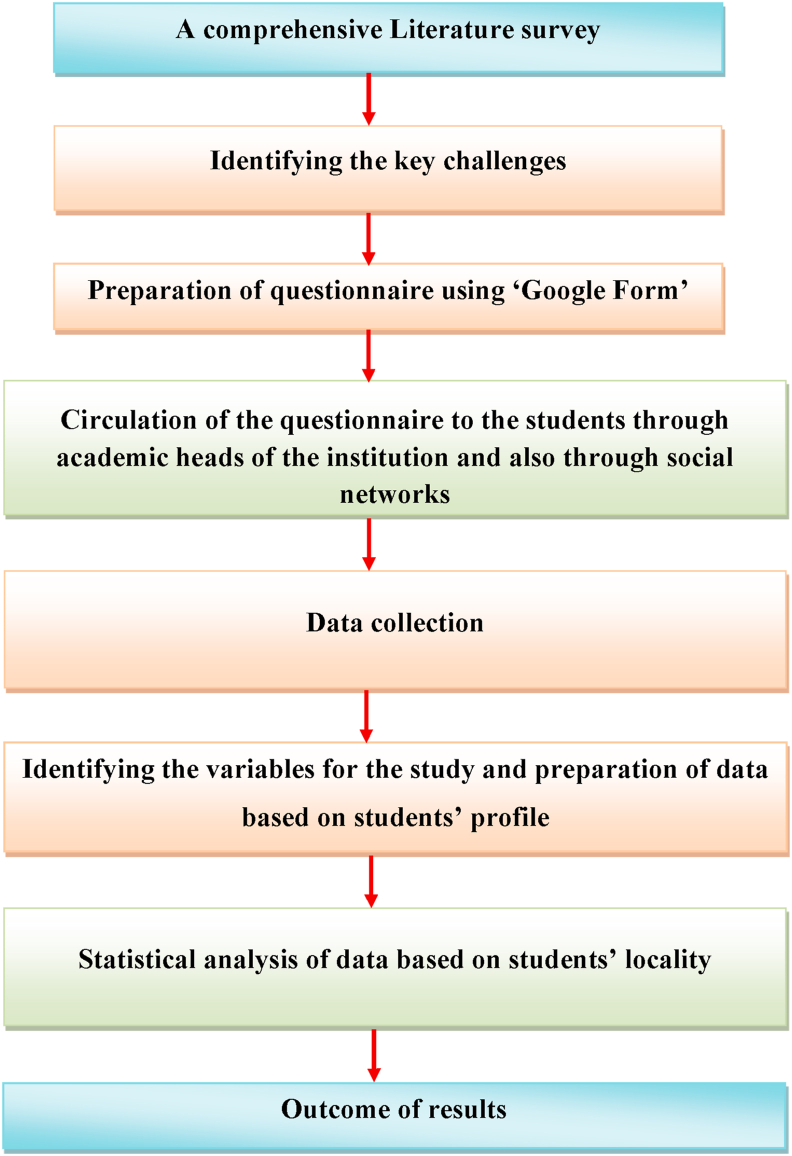
Methodology flow chart.

A survey form comprising of general awareness of COVID-19, a mental state and academic difficulty among students was created using ‘Google Form’. The students pursuing their education in higher secondary school, diploma, undergraduate, postgraduate level and research scholars who are residing in the western regions of Uganda are allowed to participate in this study. The non-Ugandan students who are studying in this region are also allowed without any gender bias. The questionnaire was shared among the students with the help of their school academic heads and through social networks. The design of the questionnaire was made simple such that the students can respond by utilizing their smart gadgets or a personal computer from their residing places. The methodology flow chart is shown in Figure 1.

### 2.1 Data collection

The western part of Uganda comprises 26 districts and the survey was conducted in those regions employing a simple random sampling technique. A total of 405 students have participated in this survey, of that 60.2% are male students, 39.3% of students are female students and 0.5% of students have not preferred to say their gender. In this study, 52.8% of Ugandan national students have participated and the rest 47.2% are foreign students who are pursuing their studies in this locality and also the students were classified according to their age group and education level. This survey was started in April 2020 and data were collected till May 2020. The data collected for this study were analyzed by the ‘Descriptive Statistical’ method among the population (N = 405) using SPSS Version 20 software. The response rate for this questionnaire was 100% and the questions in the questionnaire were given codes with a minimum of 1 and to a maximum of 5. To detail this study, total students (N = 405) participated in this survey is further classified based on their localities such as rural, semi-urban, urban and the corresponding variables age, gender, nationality and education level, the details are summarized in Table 1.

**Table 1:** Demographic Profile of student participants (N = 405).

Variables	Rural (N_1_ = 253) Number %	Semi-Urban (N_2_ = 59) Number %	Urban (N_3_ = 93) Number %	Total (N = 405) Number %
**Age**				
<18 Years	0 (0.0%)	0 (0.0%)	2 (2.2%)	2 (0.5%)
18–25 Years	169 (66.8%)	24 (40.7%)	40 (43.0%)	233 (57.5%)
26–35 Years	64 (25.3%)	18 (30.5%)	29 (31.2%)	111 (27.4%)
36–45 Years	15 (5.9%)	13 (22.0%)	13 (14.0%)	41 (10.1%)
>45 Years	5 (2.0%)	4 (6.8%)	9 (9.7%)	18 (4.4%)
**Gender**				
Male	146 (57.7%)	41 (69.5%)	57 (61.3%)	244 (60.2%)
Female	106 (41.9%)	17 (28.8%)	36 (38.7%)	159 (39.3%)
Prefer not to say	1 (0.4%)	1 (1.7%)	0 (0.0%)	2 (0.5%)
**Nationality**				
Ugandan	176 (69.6%)	14 (23.7%)	24 (25.8%)	214 (52.8%)
Non Ugandan	77 (30.4%)	45 (76.3%)	69 (74.2%)	191 (47.2%)
**Education Level**				
Higher Secondary School	2 (0.8%)	0 (0.0%)	3 (3.2%)	5 (1.2%)
Diploma	67 (26.5%)	4 (6.8%)	3 (3.2%)	74 (18.3%)
Under Graduate	104 (41.1%)	22 (37.3%)	35 (37.6%)	161 (39.8%)
Post Graduate	51 (20.2%)	14 (23.7%)	23 (24.7%)	88 (21.7%)
Research Scholar	29 (11.5%)	19 (32.2%)	29 (31.2%)	77 (19.0%)

## 3 Results and discussions

This study was classified into three sections; in the first section of this survey, nine questions were asked to ensure the general awareness about this pandemic caused by COVID-19 and few crucial questions such as the economic disaster in the country and self-sustainability were also raised. In the second section of this survey, the mental state of the students during the lockdown period due to the COVID-19 pandemic was assessed based on ten questions. In the third section of this survey, the academic difficulties facing by the students during the COVID-19 outbreak were assessed based on nine questions.

### 3.1 General awareness about COVID-19 outbreak

Humans are social beings who depend upon other individuals to fulfill their psychological, social, emotional and survival needs (Tay and Diener, 2011). In basic terms, social support means having friends, family and significant others who can provide tangible or intangible support at times of crises (Kushlev et al., 2017). The government of Uganda is striving hard to control the spread rate of COVID-19 through the suspension of human activities (lockdown) and creating awareness among the people about this COVID-19 pandemic. This section of the survey reflects the consciousness of the students residing in the western region of Uganda about the COVID-19 pandemic. The survey results are presented in Table 2.

**Table 2:** General awareness about COVID-19 outbreak.

Item 1.1	What is the main source of knowledge about COVID-19?	Media (TV, Radio, etc.)			Daily newspaper			Social media (Facebook, Twitter, WhatsApp, etc.)				Scholarly articles	Others
		288 (71.1%)			8 (2%)			99 (24.4%)				6 (1.5%)	4 (1%)
Item 1.2	Are you aware of the terms ‘communicable disease’, ‘pandemic’ and ‘epidemic’?					Yes						No	
						391 (96.5%)						14 (3.5%)	
Item 1.3	In which category COVID-19 falls?			Communicable disease			Pandemic			Epidemic			All the above
				23 (5.7%)			184 (45.4%)			6 (1.5%)			192 (47.4%)
Item 1.4	How does COVID-19 spread?			From person to person through small droplets from the nose or mouth			From surfaces around the infected person such as tables, doorknobs, and handrails			Close contact with an infected person			All the above
				43 (10.6%)			9 (2.2%)			26 (6.4%)			327 (80.7%)
Item 1.5	The most efficient defense against COVID-19 is			Taking immune-boosting foods			Social distancing			Strictly obeying the government regulations			All the above
				17 (4.2%)			63 (15.6%)			34 (8.4%)			291 (71.9%)
Item 1.6	What food is advised to increase immunity?		Carbohydrates, Protein, Fat			Vegetables, Fruits, Dry fruits			Herbs		All the above		Other
			16 (4%)			55 (13.6%)			17 (4.2%)		313 (77.3%)		4 (0.9%)
Item 1.7	Do you think the COVID-19 crisis caused economic disaster in the country?					Yes					No		
						400 (98.8%)					5 (1.2%)		
Item 1.8	Are you aware of the term self-sustainability?					Yes, I am aware					No, I am not aware		
						396 (97.8%)					9 (2.2%)		
Item 1.9	Do you believe self-sustainability as a choice for survival during this COVID-19 crisis?					Yes, I believe					No, it is not possible		
						382 (94.3%)					23 (5.7%)		

The results obtained in the first section of this survey are summarized based on the students' locality. The significance of each question among the students’ localities was deliberated with P-Value<0.05. The results are presented in Table 3.

**Table 3:** Statistical populations’ answer on general awareness among students about COVID-19 outbreak.

Questions & Locality		N	Mean	Standard Deviation	Standard Error	Minimum	95% Confidence Interval for Mean		Maximum	P-Value
							Lower Bound	Upper Bound		
Item 1.1	Rural	253	1.4506	0.90113	0.05665	1.00	1.3390	1.5622	5.00	0.000∗
	Semi-Urban	59	2.0678	1.04822	0.13647	1.00	1.7946	2.3410	4.00	
	Urban	93	1.6774	1.00175	0.10388	1.00	1.4711	1.8837	5.00	
	Total	405	1.5926	0.96956	0.04818	1.00	1.4979	1.6873	5.00	
Item 1.2	Rural	253	1.0277	0.16434	0.01033	1.00	1.0073	1.0480	2.00	0.600
	Semi-Urban	59	1.0508	0.22157	0.02885	1.00	0.9931	1.1086	2.00	
	Urban	93	1.0430	0.20398	0.02115	1.00	1.0010	1.0850	2.00	
	Total	405	1.0346	0.18291	0.00909	1.00	1.0167	1.0524	2.00	
Item 1.3	Rural	253	2.9684	1.04606	0.06577	1.00	2.8389	3.0979	4.00	0.189
	Semi-Urban	59	2.9153	1.10310	0.14361	1.00	2.6278	3.2027	4.00	
	Urban	93	2.7312	1.11453	0.11557	1.00	2.5016	2.9607	4.00	
	Total	405	2.9062	1.07219	0.05328	1.00	2.8014	3.0109	4.00	
Item 1.4	Rural	253	3.6561	0.82876	0.05210	1.00	3.5535	3.7587	4.00	0.060
	Semi-Urban	59	3.5085	1.08870	0.14174	1.00	3.2248	3.7922	4.00	
	Urban	93	3.3871	1.17983	0.12234	1.00	3.1441	3.6301	4.00	
	Total	405	3.5728	0.96357	0.04788	1.00	3.4787	3.6670	4.00	
Item 1.5	Rural	253	3.5613	0.79757	0.05014	1.00	3.4625	3.6600	4.00	0.051
	Semi-Urban	59	3.2881	1.06756	0.13898	1.00	3.0099	3.5663	4.00	
	Urban	93	3.3763	1.03119	0.10693	1.00	3.1640	3.5887	4.00	
	Total	405	3.4790	0.90252	0.04485	1.00	3.3909	3.5672	4.00	
Item 1.6	Rural	253	3.6759	0.78534	0.04937	1.00	3.5787	3.7731	5.00	0.012∗
	Semi-Urban	59	3.3559	1.02994	0.13409	1.00	3.0875	3.6243	5.00	
	Urban	93	3.4516	0.98374	0.10201	1.00	3.2490	3.6542	5.00	
	Total	405	3.5778	0.87992	0.04372	1.00	3.4918	3.6637	5.00	
Item 1.7	Rural	253	1.0079	0.08873	0.00558	1.00	0.9969	1.0189	2.00	0.264
	Semi-Urban	59	1.0339	0.18252	0.02376	1.00	0.9863	1.0815	2.00	
	Urban	93	1.0108	0.10370	0.01075	1.00	0.9894	1.0321	2.00	
	Total	405	1.0123	0.11056	0.00549	1.00	1.0015	1.0231	2.00	
Item 1.8	Rural	253	1.0158	0.12499	0.00786	1.00	1.0003	1.0313	2.00	0.302
	Semi-Urban	59	1.0169	0.13019	0.01695	1.00	0.9830	1.0509	2.00	
	Urban	93	1.0430	0.20398	0.02115	1.00	1.0010	1.0850	2.00	
	Total	405	1.0222	0.14759	0.00733	1.00	1.0078	1.0366	2.00	
Item 1.9	Rural	253	1.0237	0.15246	0.00959	1.00	1.0048	1.0426	2.00	0.000∗
	Semi-Urban	59	1.1525	0.36263	0.04721	1.00	1.0580	1.2470	2.00	
	Urban	93	1.0860	0.28192	0.02923	1.00	1.0280	1.1441	2.00	
	Total	405	1.0568	0.23173	0.01151	1.00	1.0342	1.0794	2.00	

∗P-Value<0.05- Highly significant.

In the first section of this survey, a question was asked about the source of knowledge on the COVID-19 outbreak. In this item majority of the students residing in rural and urban areas have answered Media (TV, Radio, etc.), whereas for the semi-urban students it was both the Broadcast Media (TV, Radio, etc.) and the Social media (Facebook, Twitter, WhatsApp,etc.). In the second and third items, questions were asked the terms ‘communicable disease’, ‘epidemic’ and ‘pandemic’ and irrespectively most of the students are aware of these terms. The fourth, fifth and sixth items are related to the spreading and preventive measures against COVID-19 and most of the students are aware of the spread and self-prevention from the disease. In the seventh item, the view of the student on the country's economy was assessed and more than 95% of the students answered that the COVID-19 outbreak causes economic disaster to the country. The eighth and ninth items are related to self-sustainability during this pandemic situation and more than 85% of the students are aware of the term self-sustainability and also they believe that it is a remedy for surveying in this pandemic situation. From the analysis it is evident that the general awareness was significantly associated with the knowledge about the COVID-19 pandemic, food habits to increase the immunity of the body and self-sustainability is a choice for survival during this pandemic crisis.

### 3.2 The mental state of a student during the COVID-19 outbreak

‘Mental state’ is a vital term encompassing physical, psychological, social, and spiritual well-being which involves one's resourcefulness and managing capabilities, the ability to be a productive and contributing member of society (Wang and Geng, 2019). Any deviance in any of these spheres of life is often considered as an issue with mental illness (Brüggen et al., 2017; Prakash and Gupta, 2019). Depression and anxiety are the common mental health disorders that affect nearly two-fifth of the surveyed population and thereby significantly contributing to a rise in the health burden (Wang et al., 2019). The results of the mental state of a student residing in the western regions of Uganda during the COVID-19 outbreak are summarized in Table 4 and its statistical results are given in Table 5.

**Table 4:** The mental state of a student during the COVID-19 outbreak.

Item 2.1	Due to this continuous lockdown, which of the following is highly affected in your life?	Education		Family income				Entertainment				All the above
		58 (14.3%)		65 (16%)				8 (2%)				274 (67.7%)
Item 2.2	Whose role is significant during this pandemic situation?	Doctor, Nurse, Police, Health Workers and Volunteers		Ministers, District Administrators, Teachers				Family, Friends, Relatives				All the above
		201 (49.6%)		2 (0.5%)				5 (1.2%)				197 (48.6%)
Item 2.3	How long have you been staying at home?	0–10 Days		10–20 Days				20–30 Days				30 Days and above
		1 (0.3%)		12 (3%)				3 (0.7%)				389 (96%)
Item 2.4	Do you have fear about this pandemic situation due to COVID-19?	Yes		No				Some times				Not willing to share
		179 (44.2%)		45 (11.1%)				135 (33.3%)				46 (11.4%)
Item 2.5	Which of the following ease your anxiety about COVID-19?	Reading books, writing articles		Workouts and Meditation				Talking with others				All the above
		67 (16.5%)		40 (9.9%)				44 (10.9%)				254 (62.7%)
Item 2.6	Due to this Lockdown, is your family affected economically?			Yes					No			
				376 (92.8%)					29 (7.2%)			
Item 2.7	If your parent is a daily wager or employee, how did they get the money during this pandemic situation?	Workplace		Government		Self-employment			Borrowing from others		Other	
		117 (28.9%)		39 (9.6%)		135 (33.3%)			95 (23.5%)		19 (4.7%)	
Item 2.8	Do you think media/social media is making you panic?		Yes				No					
			323 (79.8%)				82 (20.2%)					
Item 2.9	What is your learning from this lockdown period due to COVID-19?	Self-sustainability		Humanity	Cleanliness			All the above		Other		
		40 (9.9%)		13 (3.2%)	22 (5.4%)			328 (81%)		2 (0.5%)		
Item 2.10	How are you spending your time during this lockdown period due to COVID-19?	Reading books, online education		Workouts and Meditation				Spending time with family members, chatting with friends				All the above
		78 (19.3%)		13 (3.2%)				42 (10.4%)				272 (67.2%)

**Table 5:** Statistical populations’ answer to the mental state of the students during the COVID-19 outbreak.

Questions & Locality		N	Mean	Standard Deviation	Standard Error	Minimum	95% Confidence Interval for Mean		Maximum	P-Value
							Lower Bound	Upper Bound		
Item 2.1	Rural	253	3.4506	1.05904	0.06658	1.00	3.3195	3.5817	4.00	0.000∗
	Semi-Urban	59	2.8475	1.28403	0.16717	1.00	2.5128	3.1821	4.00	
	Urban	93	2.8710	1.22675	0.12721	1.00	2.6183	3.1236	4.00	
	Total	405	3.2296	1.16641	0.05796	1.00	3.1157	3.3436	4.00	
Item 2.2	Rural	253	2.8696	1.44570	0.09089	1.00	2.6906	3.0486	4.00	0.000∗
	Semi-Urban	59	1.7119	1.26014	0.16406	1.00	1.3835	2.0403	4.00	
	Urban	93	1.9462	1.39382	0.14453	1.00	1.6592	2.2333	4.00	
	Total	405	2.4889	1.49023	0.07405	1.00	2.3433	2.6345	4.00	
Item 2.3	Rural	253	3.9644	0.25727	0.01617	2.00	3.9326	3.9963	4.00	0.030∗
	Semi-Urban	59	3.8644	0.54004	0.07031	1.00	3.7237	4.0051	4.00	
	Urban	93	3.8602	0.50198	0.05205	2.00	3.7568	3.9636	4.00	
	Total	405	3.9259	0.37817	0.01879	1.00	3.8890	3.9629	4.00	
Item 2.4	Rural	253	2.2174	1.15649	0.07271	1.00	2.0742	2.3606	4.00	0.042∗
	Semi-Urban	59	1.8475	0.88695	0.11547	1.00	1.6163	2.0786	4.00	
	Urban	93	2.0215	1.05272	0.10916	1.00	1.8047	2.2383	4.00	
	Total	405	2.1185	1.10390	0.05485	1.00	2.0107	2.2264	4.00	
Item 2.5	Rural	253	3.3636	1.09932	0.06911	1.00	3.2275	3.4998	4.00	0.001∗
	Semi-Urban	59	2.9661	1.24521	0.16211	1.00	2.6416	3.2906	4.00	
	Urban	93	2.8925	1.20201	0.12464	1.00	2.6449	3.1400	4.00	
	Total	405	3.1975	1.16274	0.05778	1.00	3.0839	3.3111	4.00	
Item 2.6	Rural	253	1.0711	0.25758	0.01619	1.00	1.0393	1.1030	2.00	0.716
	Semi-Urban	59	1.0508	0.22157	0.02885	1.00	0.9931	1.1086	2.00	
	Urban	93	1.0860	0.28192	0.02923	1.00	1.0280	1.1441	2.00	
	Total	405	1.0716	0.25815	0.01283	1.00	1.0464	1.0968	2.00	
Item 2.7	Rural	253	2.5850	1.19759	0.07529	1.00	2.4367	2.7333	5.00	0.289
	Semi-Urban	59	2.8644	1.23815	0.16119	1.00	2.5417	3.1871	5.00	
	Urban	93	2.6774	1.36064	0.14109	1.00	2.3972	2.9576	5.00	
	Total	405	2.6469	1.24331	0.06178	1.00	2.5255	2.7684	5.00	
Item 2.8	Rural	253	1.0988	0.29900	0.01880	1.00	1.0618	1.1358	2.00	0.000∗
	Semi-Urban	59	1.4407	0.50073	0.06519	1.00	1.3102	1.5712	2.00	
	Urban	93	1.3333	0.47396	0.04915	1.00	1.2357	1.4309	2.00	
	Total	405	1.2025	0.40234	0.01999	1.00	1.1632	1.2418	2.00	
Item 2.9	Rural	253	3.6798	0.87529	0.05503	1.00	3.5715	3.7882	5.00	0.024∗
	Semi-Urban	59	3.5593	0.95179	0.12391	1.00	3.3113	3.8074	4.00	
	Urban	93	3.3656	1.12080	0.11622	1.00	3.1348	3.5964	5.00	
	Total	405	3.5901	0.95435	0.04742	1.00	3.4969	3.6833	5.00	
Item 2.10	Rural	253	3.3439	1.13928	0.07163	1.00	3.2028	3.4849	4.00	0.133
	Semi-Urban	59	3.0508	1.27879	0.16648	1.00	2.7176	3.3841	4.00	
	Urban	93	3.1398	1.24753	0.12936	1.00	2.8829	3.3967	4.00	
	Total	405	3.2543	1.18865	0.05906	1.00	3.1382	3.3704	4.00	

∗P-Value<0.05- Highly significant.

COVID-19 outbreak affects most of the students' daily life. In the second section of this survey, the first question is about the effect of routine life due to continuous lockdown. The students responded irrespective of the locality is family income and education are highly affected. In the second item, more than 65% of the students from semi-urban and urban areas answered the role of doctor, nurse, police, health workers and volunteers are significant in this pandemic crisis whereas 37% of students from rural areas answered the same. But 61% of students from rural regions answered that the role of ruling administrators and the beloved ones along with the health workers are significant in this COVID-19 outbreak. More than 92% of students from all the regions answered that they are staying at home for more than 30days in this pandemic situation. The fourth item assessed the daringness of the students about this COVID-19 outbreak. More than 43% of students from all the regions have fear of this disease and more than 27% of students from all the regions sometimes have fear and less than 25% of students reported that they are fearless about this disease and at the maximum of 15% of students responded not interested to share their opinion. In the fifth item, a question was raised to the students about how they ease out their anxiety in this pandemic situation. For this 72% of the students from rural regions answered that they read, write, do workouts, meditate and share their thoughts with the loved ones whereas 53% of students from semi-urban and 45% of students from the urban region also do the same. The sixth and seventh items of this section are about the family's economic situation. It is crucial to note that more than 90% of the students in all the regions responded that this COVID-19 outbreak affects the economic status of their family and about 10% ignored it and most of the students from rural regions responded that their parents receive or earn money from their regular work and earn by self-employment in this outbreak whereas, in case of the semi-urban and urban region, students responded that their parents even borrow money from others or take a loan for the survival of their family. In the eight-item, a question was raised on the role of media/social media in this pandemic situation. It is noted that 90% of the students residing in rural regions responded that the media/social media is making them panic about this disease whereas in semi-urban and urban it is 56% and 67% of students reported that media/social media is making them panic about this disease and almost about 44% in semi-urban and 33% in urban ignored this matter. In the ninth and tenth items of this section, questions were asked for learning from this lockdown and spending the time by a student during this lockdown due to COVID-19. Most of the students from all the regions responded that they learned self-sustainability, humanity and cleanliness from this pandemic situation and also most of the students spend their time reading, writing, workouts, meditation, and spending time with their loved ones in this pandemic situation. The results reveal that the mental state of the students significantly correlated with their routine life, the role of personalities during this pandemic crisis, staying at home due to lockdown, fear about this pandemic, anxiety and learning from this crisis.

### 3.3 Academic difficulties among the students during the COVID-19 outbreak

Education is a gradual process of acquiring critical thinking, capabilities and expertise which helps the individual to attain personal goals and work productively for the betterment of mankind (Petek and Bedir, 2018; Abubakar et al., 2019). The school and college students all over the world are facing serious challenges due to this COVID-19 pandemic. The regular academic and research activities of students in Africa is distorted largely due to the COVID-19 pandemic. The results of the academic difficulties faced by the students of western Uganda in this COVID-19 pandemic crisis are summarized in Table 6 and its statistical results are given in Table 7.

**Table 6:** Academic difficulties during the COVID-19 outbreak.

Item 3.1	Have you got your syllabus completed for this academic year?	Yes	No
		149 (36.8%)	256 (63.2%)
Item 3.2	Are you studying your subjects during this lockdown period?	Yes	No
		368 (90.9%)	37 (9.1%)
Item 3.3	Do your current studies get affected during this lockdown period?	Yes	No
		361 (89.1%)	44 (10.9%)
Item 3.4	If the lockdown is extended, will you submit your course works through online?	Yes	No
		364 (89.9%)	41 (10.1%)
Item 3.5	In what mode, you are willing to write your end semester or year-end examination?	Regular mode (Physically present after the lockdown)	Online mode
		318 (78.5%)	87 (21.5%)
Item 3.6	If the examination is conducted through online mode, do you have the facilities in your locality?	Yes	No
		156 (38.5%)	249 (61.5%)
Item 3.7	Are you aware of the open book examination system?	Yes	No
		349 (86.2%)	56 (13.8%)
Item 3.8	Are you learning your subjects through online mode during this lockdown period?	Yes	No
		235 (58%)	170 (42%)
Item 3.9	Do you feel this lockdown affected your career opportunities?	Yes	No
		352 (86.9%)	53 (13.1%)

**Table 7:** Statistical populations’ answer on the academic difficulties among students during the COVID-19 outbreak.

Questions & Locality		N	Mean	Standard Deviation	Standard Error	Minimum	95% Confidence Interval for Mean		Maximum	P-Value
							Lower Bound	Upper Bound		
Item 3.1	Rural	253	1.7668	0.42371	0.02664	1.00	1.7143	1.8193	2.00	0.000∗
	Semi-Urban	59	1.4407	0.50073	0.06519	1.00	1.3102	1.5712	2.00	
	Urban	93	1.3871	0.48973	0.05078	1.00	1.2862	1.4880	2.00	
	Total	405	1.6321	0.48283	0.02399	1.00	1.5849	1.6793	2.00	
Item 3.2	Rural	253	1.0632	0.24388	0.01533	1.00	1.0330	1.0934	2.00	0.023∗
	Semi-Urban	59	1.1695	0.37841	0.04926	1.00	1.0709	1.2681	2.00	
	Urban	93	1.1183	0.32469	0.03367	1.00	1.0514	1.1851	2.00	
	Total	405	1.0914	0.28847	0.01433	1.00	1.0632	1.1195	2.00	
Item 3.3	Rural	253	1.0553	0.22909	0.01440	1.00	1.0270	1.0837	2.00	0.000∗
	Semi-Urban	59	1.2203	0.41803	0.05442	1.00	1.1114	1.3293	2.00	
	Urban	93	1.1828	0.38859	0.04030	1.00	1.1028	1.2628	2.00	
	Total	405	1.1086	0.31157	0.01548	1.00	1.0782	1.1391	2.00	
Item 3.4	Rural	253	1.1067	0.30937	0.01945	1.00	1.0684	1.1450	2.00	0.130
	Semi-Urban	59	1.1525	0.36263	0.04721	1.00	1.0580	1.2470	2.00	
	Urban	93	1.0538	0.22677	0.02352	1.00	1.0071	1.1005	2.00	
	Total	405	1.1012	0.30201	0.01501	1.00	1.0717	1.1307	2.00	
Item 3.5	Rural	253	1.1383	0.34594	0.02175	1.00	1.0955	1.1812	2.00	0.000∗
	Semi-Urban	59	1.2881	0.45678	0.05947	1.00	1.1691	1.4072	2.00	
	Urban	93	1.3763	0.48709	0.05051	1.00	1.2760	1.4767	2.00	
	Total	405	1.2148	0.41120	0.02043	1.00	1.1746	1.2550	2.00	
Item 3.6	Rural	253	1.7708	0.42118	0.02648	1.00	1.7186	1.8229	2.00	0.000∗
	Semi-Urban	59	1.4407	0.50073	0.06519	1.00	1.3102	1.5712	2.00	
	Urban	93	1.3011	0.46121	0.04783	1.00	1.2061	1.3961	2.00	
	Total	405	1.6148	0.48724	0.02421	1.00	1.5672	1.6624	2.00	
Item 3.7	Rural	253	1.1028	0.30426	0.01913	1.00	1.0651	1.1404	2.00	0.023∗
	Semi-Urban	59	1.2203	0.41803	0.05442	1.00	1.1114	1.3293	2.00	
	Urban	93	1.1828	0.38859	0.04030	1.00	1.1028	1.2628	2.00	
	Total	405	1.1383	0.34561	0.01717	1.00	1.1045	1.1720	2.00	
Item 3.8	Rural	253	1.5810	0.49437	0.03108	1.00	1.5198	1.6422	2.00	0.000∗
	Semi-Urban	59	1.2203	0.41803	0.05442	1.00	1.1114	1.3293	2.00	
	Urban	93	1.1075	0.31146	0.03230	1.00	1.0434	1.1717	2.00	
	Total	405	1.4198	0.49413	0.02455	1.00	1.3715	1.4680	2.00	
Item 3.9	Rural	253	1.0711	0.25758	0.01619	1.00	1.0393	1.1030	2.00	0.000∗
	Semi-Urban	59	1.2203	0.41803	0.05442	1.00	1.1114	1.3293	2.00	
	Urban	93	1.2366	0.42727	0.04431	1.00	1.1486	1.3246	2.00	
	Total	405	1.1309	0.33767	0.01678	1.00	1.0979	1.1638	2.00	

∗P-Value<0.05- Highly significant.

The learning habits of the students might vary during holidays when compared with the regular academic year. As of now the COVID-19 induced lockdown made the student community stay in the home for many months. The first item in the third section of the survey, a question was raised regarding the syllabus completion for the courses taken by the students for this academic year. Most of the students residing in rural regions reported that the syllabus is not completed whereas more than 55% of students in semi-urban and urban regions reported that their course syllabus was completed for this academic year and more than 83% of students from all the regions answered that they are learning their courses during this lockdown period due to COVID-19 outbreak. Also, it is crucial to note that regular academic studies are affected for more than 78% of students from all the regions. If the lockdown extends, more than 85% of the students accepted to submit their coursework/assignment through online mode. But most of the students from all the regions are not willing to write their academic end semester examination through online mode because they don't have enough facility in their locality. It is critical to watch that more than 75% of students from all the regions feel this pandemic situation due to COVID-19 affect their career opportunities. The results reveal that the students' problems in their academics such as syllabus coverage, learning subjects during the lockdown, worry about the current study, facilities and willingness of online end-semester examination, awareness of open-book exams, learning subjects through online and career opportunities were significantly correlated with the academic difficulties among the students' localities.

## 4 Limitations of the study

The researchers discovered few constraints in this investigation. Initially, the research instrument used was a questionnaire and the outcome depends on the awareness of the students. This survey was directed to gather information for the perceptive level of students about COVID-19, mental state and academic difficulties faced by the students during this pandemic crisis. In this way, the information gathered was considered as self-revealed information and it depends on the ability of the student to answer. The researchers interpret the data comparing to what message that the respondents want to convey. Secondly, this survey was conducted in the western part of Uganda. The other parts of Uganda were not involved in this study. Therefore the results focus on only the western side not diverse enough to provide a full-frame of Uganda.

## 5 Conclusion

This study emphasizes the general awareness related to the COVID-19 crisis, the mental state of the students and the academic problems faced by the students residing in the western regions of Uganda. Most of the students are aware of this pandemic crisis and they believe that self-sustainability is a choice for survival during this crisis. The significance level of self-sustainability is high among the students residing in different localities such as rural, semi-urban and urban. Due to this continuous lockdown, the students are staying at home for more than one month and they are worried about their family income. They are also facing problems in their academics due to the shutdown of educational institutions. Their regular studies distorted largely in this pandemic crisis and they registered their opinion that they don't have internet or other facilities in their localities to continue their studies via online. They feel that this continuous lockdown would affect their carrier opportunities badly.

## Declarations

### Author contribution statement

A. Meji M: Conceived and designed the experiments; Performed the experiments; Analyzed and interpreted the data; Wrote the paper.

M. S. Dennison: Contributed reagents, materials, analysis tools or data; Wrote the paper.

### Funding statement

This research did not receive any specific grant from funding agencies in the public, commercial, or not-for-profit sectors.

### Declaration of interests statement

The authors declare no conflict of interest.

### Additional information

No additional information is available for this paper.
